# Uncertain of uncertainties? A comparison of uncertainty quantification metrics for chemical data sets

**DOI:** 10.1186/s13321-023-00790-0

**Published:** 2023-12-18

**Authors:** Maria H. Rasmussen, Chenru Duan, Heather J. Kulik, Jan H. Jensen

**Affiliations:** 1 Department of Chemistry, University of Copenhagen, Copenhagen, Denmark; 2 Department of Chemical Engineering, Massachusetts Institute of Technology, Cambridge, USA; 3 Department of Chemistry, Massachusetts Institute of Technology, Cambridge, USA

## Abstract

With the increasingly more important role of machine learning (ML) models in chemical research, the need for putting a level of confidence to the model predictions naturally arises. Several methods for obtaining uncertainty estimates have been proposed in recent years but consensus on the evaluation of these have yet to be established and different studies on uncertainties generally uses different metrics to evaluate them. We compare three of the most popular validation metrics (Spearman’s rank correlation coefficient, the negative log likelihood (NLL) and the miscalibration area) to the error-based calibration introduced by Levi et al. (*Sensors* **2022**, *22*, 5540). Importantly, metrics such as the negative log likelihood (NLL) and Spearman’s rank correlation coefficient bear little information in themselves. We therefore introduce reference values obtained through errors simulated directly from the uncertainty distribution. The different metrics target different properties and we show how to interpret them, but we generally find the best overall validation to be done based on the error-based calibration plot introduced by Levi et al. Finally, we illustrate the sensitivity of ranking-based methods (e.g. Spearman’s rank correlation coefficient) towards test set design by using the same toy model ferent test sets and obtaining vastly different metrics (0.05 vs. 0.65).

**Supplementary Information:**

The online version contains supplementary material available at 10.1186/s13321-023-00790-0.

## Introduction

Machine learning applied to the chemical sciences has proved itself an important new tool for chemists and in particular computational chemists. The reported test error in chemical regression tasks is often similar or lower than for more computational demanding tasks such as DFT, making it attractive for especially high-throughput screening studies. For data driven methods such as machine learning there is a strong dependency on the training data distribution. With the vast and diverse nature of chemical space, a model with the same low error across chemical space is currently not realistic. Therefore, attention within the chemical machine learning community has lately turned towards quantifying the uncertainty on property predictions made by machine learning methods [1–7].

An important aspect of uncertainty quantification (UQ) methods is how to evaluate the performance of the uncertainty predictions made by a method. Here, it is relevant to consider some of the typical applications of the uncertainty estimates. Machine learning models are often created to be employed in high-throughput screening studies where the goal is to end up with a few candidate molecules with high probability of being good at whatever they where optimized towards. For this application, focus will be on the low uncertainties being adequately described with a significantly lower expected error, increasing the probability that predictions for the final candidates are correct. Another important application is sequential learning strategies such as Bayesian optimization and active learning, where the uncertainty estimates in conjunction with the predicted property are used to guide the choice of the next pool of training molecules. Since the uncertainty estimate associated with a molecule is directly linked to its probability of being picked, here the priority would be a decent performance across the range of uncertainties, especially the large ones.

Several studies assessing the performance of different UQ methods exist, see e.g. [3, 4, 7, 8]. Such comparison studies are challenged by the fact that the true uncertainties are generally not available. Rather the UQ methods are evaluated based on a single error-observation for each predicted uncertainty. Since no obvious evaluation metric exists, different studies use different evaluation metrics. Three of the popular evaluation metrics are Spearman’s rank correlation coefficient, the miscalibration area and the negative log likelihood (NLL). Some studies employing UQ estimates use all three evaluation metrics [3, 9] while some use one of them [8, 10, 11]. However, as pointed out by Hirschfeld et al. [3] in their benchmark study comparing a multitude of UQ methods on several datasets these three evaluation metrics do not necessarily agree on which UQ estimates are better.

Another option for evaluating the UQ estimates is based on the error-based calibration recently proposed by Levi et al. [12]. While application of this metric has seen some adoption within UQ for molecular machine learning [4, 6], there has been no head-to-head comparison of the error-based calibration metric versus the three above-mentioned popular choices applied to chemical datasets. That is what we do here. Through examples using two different chemical datasets and three different UQ methods (ensemble with random forest (RF), the latent space distance [1] and evidential regression [13]) we demonstrate the superiority of evaluating uncertainty estimates based on error-based calibration and point to the drawbacks of the three popular UQ evaluation metrics; Spearman’s rank correlation coefficient, the miscalibration area and the NLL.

## Methods

### Models

Model details are collected in the supporting information, but here we present a short overview. We use a series of ML models to predict Crippen logP [14] from a recent study [15] combined with different UQ methods for obtaining uncertainty estimates. Here, we test two kinds of models: random forest (RF) trained on ECFP4 fingerprints and graph convolutional neural networks (GCNNs). As RF models are ensemble models, there is an intrinsic uncertainty estimate given by the standard deviation ($\sigma$) of the tree predictions. Janet et al. suggested using latent space (LS) distances to quantify uncertainty when working with deep learning models [1], which we use as uncertainty estimates for the logP GCNN models.

In addition to the logP models, we train a series of models on a vertical ionization potential (IP) data set for transition metal complexes (TMCs) calculated using B3LYP published by Duan et al. [16]. For this data set we train two kinds of models: an evidential regression model recently developed [7, 13] and a simple feed forward NN where we again use the LS uncertainties for comparison.

### Evaluation metrics

We start by briefly introducing the most popular evaluation metrics, followed by a more in-depth discussion of each. The main assumption behind UQ is that the error ($\varepsilon$) of the ML prediction ($y_p$) is random and, therefore, follows a Gaussian distribution ${\mathcal {N}}$ with standard deviation $\sigma$.

1 $$\begin{aligned} y_{p}-y = \varepsilon \sim {\mathcal {N}}(0,\sigma ^{2}) \end{aligned}$$

**Fig. 1:**
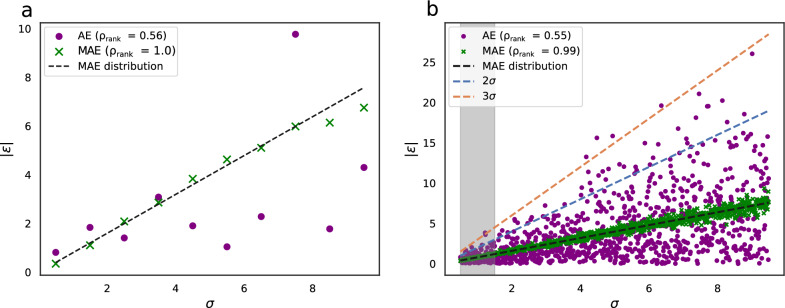
**a** Points: a single absolute error (AE) sampled from a normal distribution with standard deviation $\sigma$. x’s average absolute error (MAE) averaged over 100 points sampled from a normal distribution with standard deviation $\sigma$. The black dashed line is defined by $|\varepsilon |$ = $\sqrt{2/\pi }\sigma$ coresponding to the MAE of a Gaussian error distribution with standard deviation $\sigma$. The Spearman rank correlation coefficient is 0.56 and 1.0 for the dots and x’s, respectively. **b** Same as in (a) but in intervals of 0.01$\sigma$. The blue and orange lines are defined by $|\varepsilon | = 2\sigma$ and $|\varepsilon | = 3\sigma$, respectively. The Spearman rank correlation coefficient is 0.55 and 0.99 for the dots and x’s, respectively

Notice that this does not imply a strong correlation between $\varepsilon$ and $\sigma$ since individual random errors can fluctuate significantly. For example the Spearman rank correlation ($\rho _{rank}$) for 10 points, each randomly sampled from a normal distribution with increasing standard deviations, shown in Fig. 1a is only 0.56. More fine grained sampling (Fig. 1b) leads to a $\rho _{rank}$ of 0.55. As we will see in the results section, a more reasonable $\sigma$-range for uncertainty estimates on chemical data has the highest uncertainties roughly three times higher than the lowest uncertainties. Looking at the $\sigma$-range of 0.5$-$1.5 (grey shaded area, Fig. 1b) leads to $\rho _{rank}$ = 0.31.

**Fig. 2:**
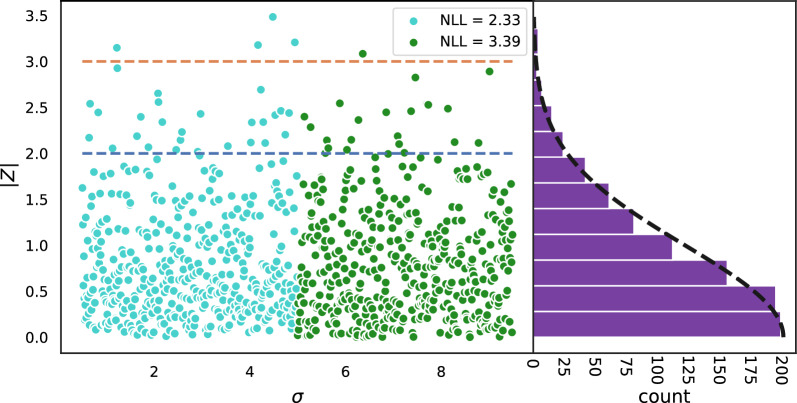
Plot of $|\varepsilon |/\sigma$ vs $\sigma$ for the points shown in Fig 1b The blue and orange lines are defined by $|Z| = |\varepsilon |/\sigma = 2$ and $|Z| = |\varepsilon |/\sigma = 3$, respectively. Though both the turquoise and green points are sampled from a normal distribution the NLL are different (2.33 and 3.39, respectively). Since the points are sampled from a normal distribution, the distribution of |*Z*| values also follow a normal distribution with a standard deviation of 1

**Fig. 3:**
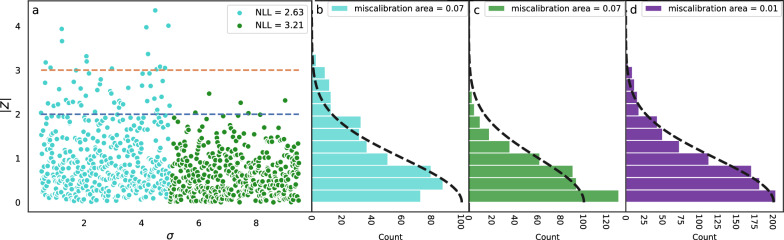
**a** Same as Fig. 2 expect that errors sampled from $\sigma < 5$ are scaled by 1.25 making the set of errors 25% too high compared to the uncertainties, $\sigma$. Similarly the errors for $\sigma > 5$ are scaled with a factor or 0.8 making these errors 20% too low based on their uncertainties. **b** The |*Z*| distribution for $\sigma < 5$ no longer follows a normal distribution with a standard deviation 1. This half of the |*Z*|-distribution has a miscalibration area of 0.07. **c** Similarly the |*Z*|-distribution for $\sigma > 5$ no longer follows a normal distribution resulting in a miscalibration area of 0.07 **d** However, the total |*Z*|-distribution still follows a normal distribution quite well with a close to zero miscalibration area due to cancellation of error between the problematic behavior of the uncertainties for $\sigma < 5$ and $\sigma > 5$

Because of the poor correlation between individual errors and the standard deviation, researchers have explored other metrics for benchmarking the relationship between uncertainty and errors. For example, if we instead plot the ratio of the random errors and the standard deviations ($|Z| = |\epsilon |/\sigma$; Fig. 2) we see that their distribution (which is independent of $\sigma$) follows a normal distribution with a standard deviation of 1. The extent to which the *Z*-distribution differs from the normal distribution can be quantified by comparing their areas, and the difference is known as the miscalibration area ($A_{mis}$; Additional file 1: Fig. S1). However, a systematic over and under estimation of the uncertainties at certain $\sigma$-ranges can lead to cancellation of errors and small values for $A_{mis}$ (Fig. 3).

The negative log likelihood (NLL) is a function of both $\sigma$ and |*Z*| (Eqn. 4) and has also been used for UQ, where lower values are considered better. However, a lower NLL does not necessarily mean better agreement between uncertainties and errors as illustrated in Figs. 2 and 3a.

**Fig. 4:**
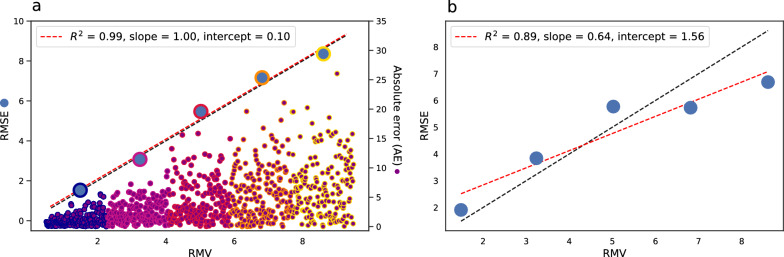
**a** Demonstration of error-based calibration. Purple dots: simulated absolute errors (same as Fig. 1b). The errors are divided into bins (here five) according to their uncertainties. The RMSE and root mean variance (RMV) of each bin of errors is calculated (blue dots). For well-calibrated uncertainties (as here), the RMSE vs. RMV plot should follow a straight line with a slope of 1 and an intercept of 0. **b** Error-based calibration for the error-uncertainty distribution in Fig. 3a which were scaled to create a mismatch between errors and uncertainties. Unlike the miscalibration area, the error-based calibration metrics catch this mismatch and we see that both linear fit, slope and intercept gets worse

The only firm correlation between random error and uncertainty is that $\sigma$ correlates with both the *average* absolute error and the root mean square error (RMSE).

2 $$\begin{aligned} \langle |\varepsilon | \rangle =\frac{1}{n} \sum _i^n |y_i^p-y| = \sqrt{\frac{2}{\pi }}\sigma \end{aligned}$$

3 $$\begin{aligned} \langle \varepsilon ^2 \rangle =\frac{1}{n} \sum _i^n (y_i^p-y)^2 = \sigma ^2 \end{aligned}$$

While *n* in principle refers to all errors, it also holds for a suitably large subset, as shown in Fig. 4, and is known as error-based calibration. As we will show in this paper, this is the superior metric for UQ validation. Below follows a more detailed description of how each metric is calculated and its interpretation.

**Spearman’s rank correlation coefficient** ($\varvec{\rho _{rank}}$) identifies the ability of the uncertainty estimate to rank the observed errors from low to high. It is calculated by giving the list of uncertainties and the list of absolute errors an integer corresponding to their magnitude i.e. the 10th lowest error gets the value 10. The normal Pearson’s correlation coefficient is then calculated for the two ranked lists consisting of integers. The idea in using ranking-based methods such as Spearman’s rank correlation coefficient to asses UQ quality is that a lower uncertainty will have a higher probability of low error compared to a higher uncertainty. However, perfect correlation ($\rho _{rank} = 1$) should not be expected since a high uncertainty can still produce a low error.

$\rho _{rank}$ does not take absolute magnitude of the uncertainties into account. For two uncertainties of similar magnitude, there is close to 50% probability that the lower uncertainty will produce a higher error, so that the probability of the uncertainties being ranked “wrong” is high. That probability decreases with increasing difference in the uncertainties. Thus, the distribution of the uncertainties has a big impact on the ability to rank errors according to uncertainties and hence what magnitude of $\rho _{rank}$ we should expect. Perhaps that also explains the differing interpretations of $\rho _{rank}$ existing in the literature. Tynes et al. found $\rho _{rank}$ ranging between 0.2 and 0.65 across tasks for their pairwise difference regression (PADRE) uncertainty estimates and used this as an indication that the uncertainty estimator is “a useful proxy for error across all tasks examined” [2]. Greenman et al. obtained $\rho _{rank} = 0.52$ for the ensemble variances and took this as suggesting that “one should not necessarily consider the rank order of the prediction uncertainties to be a good approximation of the rank ordering of the prediction errors” [17]. Hirschfeld et al. found $\rho _{rank}$ ranging between $-$0.17 and 0.34 for the lipophilicity data set when applying a range of UQ methods and concluded that “no method is able to perform particularly well” [3].

While a negative $\rho _{rank}$ indicates a problem with ones uncertainties, having obtained a positive $\rho _{rank}$ it is not clear from the number itself whether one should be alarmed or satisfied. To account for some of these drawbacks, we introduce the simulated Spearman’s rank correlation coefficient ($\rho _{rank}^{sim}$), where errors are randomly drawn from the predicted uncertainties (assuming Gaussian errors) and the $\rho _{rank}^{sim}$ is calculated based on the simulated errors. Doing this a number of times (typically 1000) we obtain an expected mean for the $\rho _{rank}$ as well as a standard deviation. The value of $\rho _{rank}^{sim}$ defines the value of $\rho _{rank}$ one *should* get for the predicted uncertainty distribution. Problems can be identified by a big discrepancy between the simulated and observed values.

Confidence curves are another popular choice of metric belonging to the ranking-based methods. The confidence curve shows the change in test set error as data points are excluded based on the predicted uncertainty (highest uncertainty points excluded first). One would then expect a decreasing curve. The observed confidence curve is often compared with what one would get for an “oracle”, which represents the quite unrealistic scenario that the ranking of the errors and uncertainties are perfectly correlated (corresponding to $\rho _{rank}=1$), meaning that the uncertainty predictor is actually an error predictor. Here we will focus on $\rho _{rank}$ to represent the ranking-based metrics but refer the reader to work by Pernot on the use of confidence curves for UQ validation, which was published while preparing this manuscript [18]. Similarly to how we propose the simulated $\rho _{rank}$ as a reference to Spearman’s rank correlation coefficient, Pernot suggests changing the reference confidence curve from an “oracle” to a probabilistic one based on errors sampled from the predicted uncertainties assuming normally distributed errors (just like we do for $\rho _{rank}^{sim}$).

**The miscalibration area** ($\varvec{A_{mis}}$) addresses the average calibration of the uncertainty estimates; is the observed distribution of errors consistent with what would be expected from the predicted uncertainty distribution? The miscalibration area is found by calculating the calibration curve, which plots the observed fraction of errors vs. the expected fraction of errors. This is done assuming the predicted uncertainties to describe a Gaussian error distribution. The errors are expressed in terms of *Z*-scores ($Z_i=\frac{\varepsilon _i}{\sigma _i}$) describing the error, $\varepsilon _i$, as a fraction of its predicted uncertainty, $\sigma _i$. Assuming the uncertainties are correct and the errors are Gaussian, the distribution of *Z*-scores should represent a Gaussian distribution with variance=1. Therefore at $|Z|>3$ we expect to see a fraction of 0.003 of the errors, at $|Z|>2$ we expect to see 0.045 of the errors and so on. Scanning through $|Z|>x$ until $x=0$ and plotting the observed vs. expected fraction of *Z*-scores results in the calibration curve (see Additional file 1: Section S1 for more details). Perfect calibration results in a diagonal line and the miscalibration area is the area between the calibration curve and the diagonal line.

Since the miscalibration metric assesses average calibration it bears no local information. Therefore one can have zero miscalibration area (perfect average calibration) even though e.g. low uncertainties are badly calibrated if the bad local calibration is cancelled by another (opposite) bad calibration at e.g. high uncertainties. Another important aspect of the miscalibration area is the assumption of normal errors. As highlighted by Pernot, such assumptions adds a fragility to the metric, since a non-zero miscalibration area can be interpreted as both a sign that the uncertainties are not calibrated or that the assumption of normal errors was wrong [5]. As an alternative, Pernot suggests the Var$(Z) \overset{?}{=}\ 1$ test which makes no assumption on the error distribution. The test consists of calculating a 95% confidence interval of Var(*Z*) using bootstrap methods and checking whether 1 is part of the interval. For the confidence intervals, we use the BC$_a$ method [19] as implemented in SciPy [20].

**The negative log likelihood (NLL)** can be used both as a loss function to minimize during training as well as a metric to evaluate the model fitness. When calculating likelihood one assumes the predicted variances ($\sigma _i^2$) to describe a Gaussian distribution of errors and multiplies the value of the probability distribution function for the observed errors ($\varepsilon _i$). The average NLL for the test set is then:

4 $$\begin{aligned} \text {NLL} = \frac{1}{2N_{test}}\sum _{i}^{N_{test}}\Big (\text {ln}(2\pi )+\text {ln}(\sigma _i^2) + \frac{\epsilon _i^2}{\sigma _i^2}\Big ) \end{aligned}$$

Given the same error distribution ({$\epsilon _i$}) but two different predicted uncertainty distributions, the uncertainties more likely to have resulted in the observed errors will have a lower NLL. However, in the context of chemical ML models, the way of obtaining property predictions (and hence errors) and the way of obtaining uncertainties typically go hand in hand. Therefore, the error distributions are typically not the same and we are not strictly comparing the uncertainties but also the accuracies of the models. Since higher accuracy generally leads to lower NLL, we can easily have two models with the more accurate model having obtained a lower NLL but still having completely random uncertainties. Another issue with the NLL is that it really only makes sense when comparing models; the number in itself is basically meaningless. For a collection of models with similar NLL it is not obvious whether all models perform very good, decent or horrible. Again, to account for some of these drawbacks, we introduce the simulated NLL, where errors are randomly drawn from the predicted uncertainties (assuming Gaussian errors) and the NLL$^{sim}$ is calculated based on the simulated errors. As for $\rho _{rank}^{sim}$, we do this a number of times (typically 1000) to obtain an expected mean for the NLL as well as a standard deviation. Discrepancy between simulated and observed NLL hints to a problem with the uncertainties.

**Error-based calibration** was originally suggested as an UQ validation metric by Levi et al. [12] and is based on the expected one-to-one relationship between the root mean square error (RMSE) for the observed errors and the root mean variance (RMV). In order to get local information about the relation between errors and uncertainties, the errors and uncertainties are ordered and binned according to their predicted uncertainty. For each bin, consisting of $N_{bin}$ samples, the RMSE and RMV is calculated:

5 $$\begin{aligned} \text {RMSE}&= \sqrt{\frac{1}{N_{bin}}\sum _i \epsilon _i^2} \nonumber \\ \text {RMV}&= \sqrt{\frac{1}{N_{bin}}\sum _i \sigma _i^2} \end{aligned}$$

Plotting RMSE vs. RMV should then produce a linear plot with slope 1 and intercept 0. As suggested by Pernot we add 95% confidence intervals to the binned RMSE values calculated by the BC$_a$ bootstrap method [5].

## Results

We demonstrate the performance and interplay of the above described UQ validation metrics on two regression tasks; the Crippen logP [14] and the vertical IP for transition metal complexes (TMCs) recently published by Duan et al. [16].

### Crippen logP

In a recent publication [15] we trained 9 random forest (RF) models on varying training set sizes (from 100 to 150k data points) and used them to show Crippen logP as a useful benchmark for atom attributions within explainable AI (XAI) on regression tasks. Since RF models have easily obtainable uncertainty estimates in the form of the variance of individual tree predictions, we reuse the models here to test the performance of the uncertainty estimates. The data set used to train the models is a 250k molecules subset of the ZINC data base which has been used in several studies [15, 21–24].

**Table 1:** RMSE and UQ evaluation metrics for the 9 RF models trained on Crippen’s logP from [15]. The simulated values, $NLL^{sim}$ and $\rho _{rank}^{sim}$ is the average of 1000 simulated sets of test errors based on the predicted uncertainties. The number in parenthesis is the standard deviation of the 1000 values

$N_{train}$	RMSE	$R^2$	*a*	*b*	$\rho _{rank}$	$\rho _{rank}^{sim}$	$A_{mis}$	NLL	NLL$^{sim}$
100	1.29	0.84	0.62	0.62	0.11	0.19 (0.01)	0.05	1.73	1.46 (0.01)
500	1.09	0.85	0.64	0.45	0.11	0.19 (0.01)	0.03	1.51	1.39 (0.01)
1000	1.01	0.85	0.55	0.45	0.10	0.19 (0.01)	0.00	1.42	1.40 (0.01)
5000	0.93	0.81	0.57	0.42	0.10	0.18 (0.01)	0.01	1.35	1.29 (0.01)
10,000	0.90	0.82	0.58	0.40	0.11	0.19 (0.01)	0.01	1.32	1.24 (0.01)
20,000	0.86	0.86	0.58	0.37	0.11	0.18 (0.01)	0.01	1.26	1.21 (0.01)
50,000	0.81	0.88	0.61	0.31	0.11	0.19 (0.01)	0.00	1.19	1.16 (0.01)
100,000	0.77	0.85	0.67	0.26	0.13	0.20 (0.01)	0.00	1.15	1.12 (0.01)
150,000	0.75	0.91	0.69	0.23	0.14	0.21 (0.01)	0.01	1.11	1.09 (0.01)

**Fig. 5:**
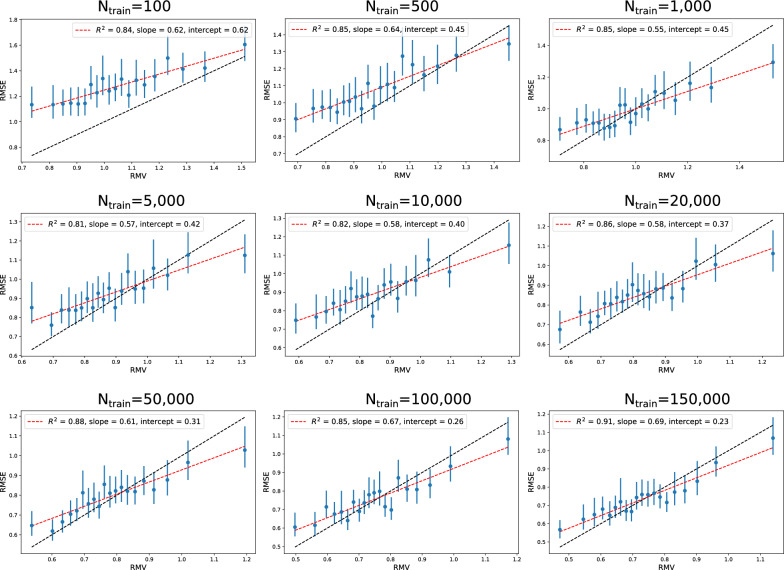
Error-based calibration plots for the uncertainties based on the nine RF models trained to predict Crippen logP. Each bin contains 250 of the test samples

Table 1 shows the test set (consisting of 5000 molecules) RMSE for the nine RF models with increasing training set size. The predictive performance clearly increases with more training data. Table 1 summarizes the UQ validation metrics described above for the RF models evaluated on the test set. The error-based calibration is quantified by three measures from the linear fit of RMSE vs. RMV; the quality of the linear fit through the $R^2$ value, the slope (*a*) and the intercept (*b*). Ideally these values should be 1, 1, and 0. The corresponding error-based calibration plots are shown in Fig. 5. Three observations are true for all models: the linear fit is decent ($R^2$ values between 0.81 and 0.91), the slope is too low (between 0.55 and 0.69) and the intercept is too high (intercept decreases from 0.62 for the $N_{train}=100$ model to 0.23 for the $N_{train}=150\text {k}$ model). Both $R^2$-value, slope and intercept is closest to the ideal value for the model trained on 150k samples but only for the intercept we see a gradual improvement as the training set size is increased. For the two models trained on the least training data ($N_{train}=100$ and $N_{train}=500$), the RMSE bins generally lie above the expected identically line. This means that the observed errors are generally higher than what would be expected based on the predicted uncertainties and thus the uncertainties are underestimated. For the remaining models we observe a trend of low uncertainties being underestimated (RMSE bins above identity line) and high uncertainties being overestimated (RMSE bins below the identity line).

All nine models show a bad agreement between $\rho _{rank}$ and $\rho _{rank}^{sim}$; the correlation between uncertainty and error is worse than expected for all models ($\rho _{rank} < \rho _{rank}^{sim}$). This is indeed what we would expect based on the error-based calibration plots; the lower-than-one slope for the error-based calibration indicates that the difference between predicted low and high uncertainties is too large. As $\rho _{rank}^{sim}$ is based on the predicted uncertainty distribution, it will return a higher number when the uncertainty distribution is predicted to be wider than what is supported by the errors.

Clearly all models have problems with local miscalibration (either under- or overestimated uncertainties). However, since the miscalibration area, $A_{mis}$, evaluates average calibration (assuming Gaussian errors) only the general miscalibration of the two models with $N_{train}=100$ and $N_{train}=500$ is caught (Table 1). The remaining seven models show close to perfect miscalibration areas. From the error-based calibration plots (Fig. 5) we see that the zero miscalibration area stems from a cancellation of the local miscalibrations: the under- and overestimated uncertainties cancel in the calculation of $A_{mis}$.

The NLL (both observed and simulated) decreases with increasing amount of training data. The trend in NLL$^{sim}$ is solely due to how the predicted uncertainty distribution changes since it does not depend on the observed errors. More accurate models means on average lower error and should be accompanied by smaller uncertainties which would result in lower NLL; this is the trend we observe in the NLL$^{sim}$. For many of the models, the observed NLL is in decent agreement with NLL$^{sim}$, i.e. we can not say that uncertainties and errors fit better for the model trained on 150,000 molecules compared to the model trained on 1000 since both have NLL $\approx$ 2 standard deviations from NLL$^{sim}$. For some (especially the models trained on 100 and 500 training samples), there is a big discrepancy between NLL and NLL$^{sim}$ which points to a problematic behaviour of the predicted uncertainties. In particular, the observed NLL is much higher than NLL$^{sim}$ indicating that the errors are generally higher than expected so the uncertainties are underestimated; we see this behavior clearly in the error-based calibration plots for $N_{train}=100$ and $N_{train}=500$ in Fig. 5.

The low $\rho _{rank}$ observed for all models could lead one to conclude that the uncertainties are useless in distinguishing high from low error. However, we do see an on average lower error for low uncertainties and likewise a higher error for high uncertainties. For the 150k model there is close to a factor 2 in difference between the lowest uncertainty bin and the highest uncertainty bin, meaning that the probability of getting a high/low error is indeed higher for a high/low uncertainty.

As suggested by Pernot [5], we also use the Var(*Z*) $\overset{?}{=}\ 1$ and the $\mu (Z)\overset{?}{=}\ 0$ test to check for average calibration and bias, respectively. The results for the RF models are summarized in Additional file 1: Table S6 and analyzed in Additional file 1: Section S5. In short, these tests also reveal problems with average calibration for the $N_{train}=100$ and $N_{train}=500$ models and these models are also seen to be biased (a tendency of overshooting the logP value). The remaining models are generally closer to being average calibrated but only the $N_{train}=1000$ model as well as the two models with most training data ($N_{train}=100$k and $N_{train}=150$k) pass the test for being average calibrated. This is contrary to the conclusions drawn based on the miscalibration area, $A_{mis}$ (Table 1) highlighting how even evaluation metrics targeting the same property of the uncertainties can lead to different conclusions.

We now turn to latent space uncertainties [1] from GCNN models, also trained on Crippen’s logP. We train two GCNN models; one with a training set with 10k data points (9k for training and 1k for validation) and one trained with 150k data points (145k for training and 5k for validation). Note that the model trained with 150k data points was published as part of the above mentioned study [15]. The 10k and 150k data sets are the same as was used for the RF models above.

The change in model from RF/ECFP4 to GCNN results in a significantly lower test set error: 0.28 for 10k and 0.16 for 150k. For the uncertainties, we test two versions of the latent space (LS). The GCNN consists of a graph convolutional network (GCN) ending in a pooling layer resulting in a vector representation of the molecule which is followed by a fully connected neural network (NN) ending in a prediction. LS-NN represents uncertainties based on the last layer of the NN and LS-GCN represents uncertainties based on the learned molecular feature vector. The latent space uncertainties are derived from the average distance in latent space of a test data point to the *k* nearest neighbors in the training set. We use $k=10$ throughout this work.

**Table 2:** UQ evaluation metrics for the latent space uncertainties of a GCNN model trained on 9k+1k data points of Crippen’s logP. For NN uncertainties, the latent space used is the very last layer of the NN. For GCN uncertainties, the latent space is the vector right after the pooling layer (LS-GCN)

LS	$R^2$	*a*	*b*	$\rho _{rank}$	$\rho _{rank}^{sim}$	$A_{mis}$	NLL	NLL$^{sim}$
NN$_{10\text {k}}$	0.31	1.12	– 0.05	– 0.04	0.07 (0.01)	0.07	0.14	0.17 (0.01)
NN$_{150\text {k}}$	0.91	1.20	– 0.03	0.17	0.24 (0.01)	0.03	– 0.50	– 0.51 (0.01)
GCN$_{10\text {k}}$	0.24	0.65	0.09	– 0.02	0.11 (0.01)	0.07	0.14	0.18 (0.01)
GCN$_{150\text {k}}$	0.85	1.85	– 0.13	0.23	0.13 (0.01)	0.05	– 0.46	– 0.45 (0.01)

**Fig. 6:**
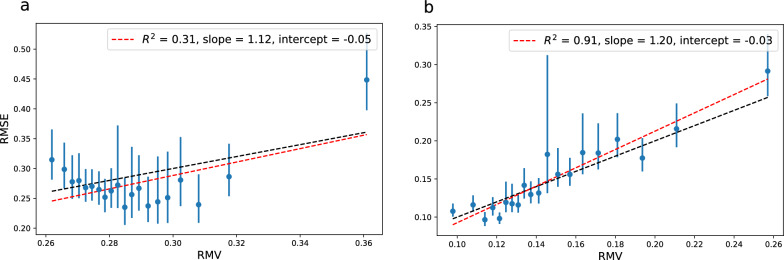
Error-based calibration plots for the LS-NN uncertainties based on a GCNN model with a 9k+1k training set consisting of Crippen’s logP training data as well as the GCNN model trained on 145k+5k samples. Each bin contains 250 of the test samples. **a** NN_10k_ $\rho _{rank}=-0.04$ **b** NN_150k_ $\rho _{rank}=0.17$

**Fig. 7:**
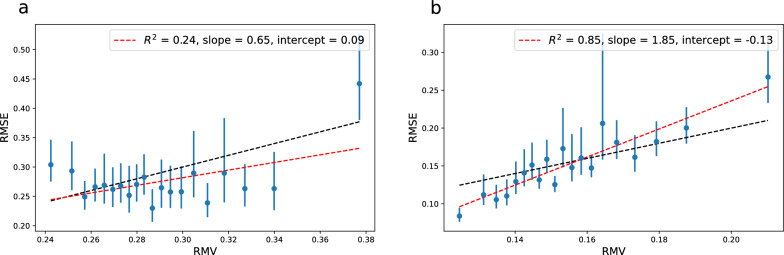
Error-based calibration plots for the LS-GCN uncertainties based on a GCNN model with a 9k+1k training set consisting of Crippen’s logP training data as well as the GCNN model trained on 145k+5k samples. Each bin contains 250 of the test samples. **a** GCN_10k_ $\rho _{rank}=-0.02$ **b** GCN150k $\rho _{rank}=0.23$

The UQ evaluation metrics for the LS-NN uncertainties and LS-GCN uncertainties including linear fit parameters for the error-based calibration are summarized in Table 2. From the error-based calibration plots (Fig. 6 for the LS-NN uncertainties and Fig. 7 for th LS-GCN uncertainties) we notice the very poor quality of the linear fit for the 9k+1k model using either LS-NN uncertainties ($R^2=0.31$) or LS-GCN uncertainties ($R^2 = 0.24$). Thus, while e.g. LS-NN uncertainties with a 10k training set show close to optimal slope (1.12) and intercept ($-$0.05), from the error-based calibration plots, we see that the 10k uncertainty estimates by no means follow the expected diagonal line (Figs. 6a and 7a). For the uncertainty bins with RMV < 0.34 the RMSE-RMV correlation seems quite random; for the LS-NN uncertainties there even seems to be a negative correlation for the lower half of the uncertainties. These observations are in agreement with the close to zero Spearman’s rank correlation coefficients observed for these uncertainty estimates (Table 2) suggesting no correlation between error and predicted uncertainty. This phenomenon of zero $\rho _{rank}$ is not uncommon; Hirschfeld et al. observed $\rho _{rank}$ values close to zero or negative for a significant part of the tested UQ methods [3]. The simulated $\rho _{rank}^{sim}$ is also quite low for the 9k+1k model using either LS-NN (0.07±0.01) or LS-GCN (0.11±0.01) uncertainties, though still significantly higher than the observed coefficient pointing to a rather narrow distribution of predicted uncertainties but also some problem with the uncertainties, which is obvious from the error-calibration plots. Thus, based on Spearman’s rank correlation coefficient we would deem these uncertainty estimates completely useless. While it is obvious that we should not use these uncertainty estimates as a way of pointing to samples with increased probability of low error, we see that the very highest uncertainties can be used as a predictor for a higher probability of high error; a detail lost by looking at a single-valued metric such as a correlation coefficient.

The performance of the uncertainty estimates changes completely for the model with 145k+5k training samples. The LS-NN$_{150\text {k}}$ uncertainties show a much better linear correlation ($R^2=0.91$) and the RMSE bins are distributed around the expected diagonal (Fig. 6b).

We also see a widening of the predicted uncertainty distribution e.g. a bigger difference between the high and low uncertainties in terms of model RMSE which fits well with the increased $\rho _{rank}^{sim}$ (0.24±0.01). While the observed $\rho _{rank}$ also increases significantly compared to the $N_{train}=$10k model ($\rho _{rank}=0.17$), there is still a discrepancy between observed and simulated $\rho _{rank}$. From the error-based calibration plot (Fig. 6b) this seems to originate from a lack of ordering within the very lowest predicted uncertainties.

The quality of the linear fit is also higher for the LS-GCN$_{150\text {k}}$ uncertainty estimates ($R^2 = 0.85$) but now with a slope almost twice as steep as the ideal. For the low uncertainties we see that the corresponding error is generally lower than expected from the uncertainty estimates; these uncertainties are overestimated. For the high uncertainties, we observe errors higher than what is expected from the uncertainty estimates; these errors are underestimated. This behavior of the uncertainty estimates is also apparent when comparing $\rho _{rank}$ with $\rho _{rank}^{sim}$. Unlike all other uncertainty estimates we have seen so far, the LS-GCN$_{150k}$ uncertainties have a higher observed $\rho _{rank}$ than its simulated $\rho _{rank}^{sim}$. As discussed for the RF uncertainties above, a predicted uncertainty distribution that is wider than what is supported by the observed errors, will result in a simulated Spearman’s rank correlation coefficient that is higher than the observed one. Similarly, a predicted uncertainty distribution that is more narrow than what fits the observed errors (as is the case here) would lead to a simulated Spearman’s rank correlation coefficient that is lower than the observed one.

Based on the $A_{mis}$ values, the average calibration is generally worse for the latent space uncertainties compared to the RF uncertainties (most severe for the model with $N_{train}$ = 10k). However, as described in the supporting information, if evaluating average calibration with the Var$(Z) \overset{?}{=}\ 1$ test, the uncertainties from the $N_{train} = 150$k model both pass (Additional file 1: Table S7).

The lower RMSE for the model with 150k training samples is also apparent in the NLL which decreases (both observed and simulated) compared to the $N_{train}=10$k model. Since for each of the two models ($N_{train}=10$k and $N_{train}=150$k) we have two sets of predicted uncertainties; LS-NN$_i$ and LS-GCN$_i$, this is a case where the error distribution is constant and differences in NLL between LS-NN$_i$ and LS-GCN$_i$ *can* be ascribed to performance of the uncertainties. For the $N_{train}=10$k model there is no difference in the NLL between LS-NN and LS-GCN uncertainties but the LS-NN$_{150\text {k}}$ uncertainties show a better model fit than the uncertainties from LS-GCN$_{150\text {k}}$ (NLL of $-$0.50 vs. $-$0.46, Table 2). This is in line with the conclusions drawn from the error-based calibration plots but opposite of a conclusion drawn solely based on which has a higher $\rho _{rank}$. The agreement between the observed and simulated NLL is decent and there is no clear trend that the observed NLL is either higher or lower than the simulated NLL. Thus, no further conclusions can be made from the NLL test.

We now investigate the LS-GCN$_{150\text {k}}$ uncertainties a bit further. In the latent space uncertainty procedure proposed by Janet et al. [1], the latent space distances (*d*) are converted to uncertainties through a linear relationship:

6 $$\begin{aligned} V(d) = \theta _0^2 + \theta _1^2\cdot d \end{aligned}$$

The parameters $\theta _0$ and $\theta _1$ are found by minimizing the NLL for the validation set. However, it seems that in this case the fitting procedure forces the uncertainties to span a too narrow range, resulting in the systematic local mis-calibrations observed in the error-based calibration plot (Fig. 7b). We test a slightly more flexible fitting procedure:

7 $$\begin{aligned} V(d) = \theta _0 + \theta _1^2\cdot d \end{aligned}$$

The only difference is the possibility of a negative offset to the linear relationship. As this change can in principle lead to negative uncertainties, which is clearly unphysical, we set $V(d)=0.0001$ if $V(d)<0$.

**Fig. 8:**
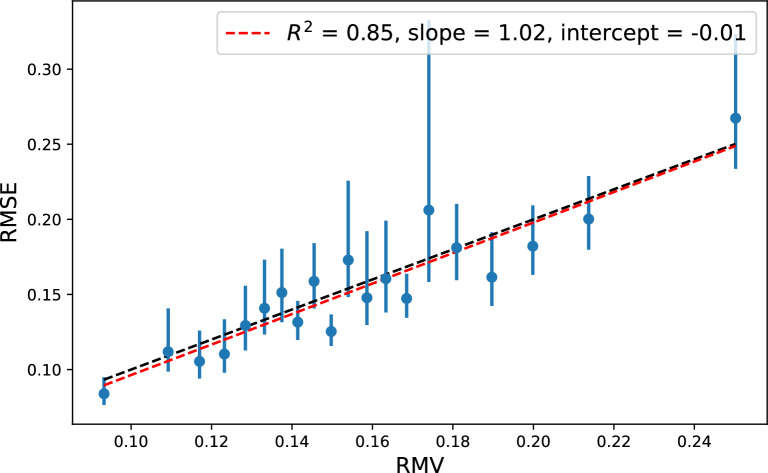
Error-based calibration plot for the LS-GCN$_{150k}$ uncertainties fitted with Eqn. 7 instead of Eqn. 6. Each bin contains 250 samples of the test set

The error-based calibration plot for the LS-GCN$_{150k}$ uncertainties fitted with Eqn. 7 is shown in Fig. 8. The local calibration is clearly much better (a similar re-calibration could have been performed for the RF uncertainties) and the NLL is lowered to $-$0.48 (NLL$^{sim}=-0.46\pm 0.01$). While the $\rho _{rank}$ does not change when doing a linear re-calibration, the simulated one is now in agreement with the observed ($\rho _{rank} = 0.23$ vs. $\rho _{rank}^{sim} = 0.24\pm 0.01$) due to the more accurate uncertainty distribution. The average calibration metrics are practically unchanged (both $A_{mis}$ and the *Z*-metrics).

Uncertainties behaving like the LS uncertainties obtained from the GCNN model with 10k logP training samples should be used with caution and the error-based calibration plots reveal the details necessary to do just that. To test whether the problematic uncertainties obtained for the 10k dataset were due to a “bad” selection of training samples, we repeat the analysis for four models trained on different 10k sets of Crippen’s logP values. Results are in the supporting information (Additional file 1: Tables S11 and S7 and Figs. S3 and S4) but are generally similar to what is presented above.

### Vertical IP

In this section we will use a data set more representative of a typical ML dataset within chemical machine learning. Duan et al. recently published a data set of vertical ionization potential (IP) calculations for transition metal complexes (TMCs) with a range of different DFT functionals [16]. The RAC-155 features developed by the Kulik group [25] are used to represent the transition metal complexes (TMCs) from which a ML model for each functional is trained and used to obtain consensus predictions. Here, we exemplify the use of the above described evaluation metrics by training some simple feed-forward NNs in PyTorch [26] on the B3LYP data set (see SI for model details). We train five simple feed-forward NNs as well as five evidential NNs following Amini et al. [13] and Soleimany et al. [7]. The evidential NN is designed to predict four parameters ($\gamma , \nu , \alpha$ and $\beta$) for each data sample defining an evidential distribution from which a mean prediction as well as predictions for both aleatoric and epistemic uncertainty can be obtained (see Additional file 1: Section S2 for details).

Five different models are trained with different random splits (train_test_split from scikit-learn [27] with different random states) of the training data into training (80%) and validation (20%) sets. The test set RMSE ranges between 0.55$-$0.65 eV (Additional file 1: Table S12). Here we focus on a single set of uncertainties (for the model trained with random seed 42), but results for the remaining models can be found in the supporting information.

**Table 3:** EA: UQ evaluation metrics for the evidential uncertainties of an evidential NN model trained on the vertical IP dataset. LS flex: UQ evaluation metrics for the LS flex uncertainties of one of the five feed forward NN models trained with different random splits of the vertical IP training data. Random seed 42 is used in both cases

UQ	$R^2$	*a*	*b* (eV)	$\rho _{rank}$	$\rho _{rank}^{sim}$	$A_{mis}$	NLL	NLL$^{sim}$
EV	0.83	0.27	0.35	0.41	0.56(0.05)	0.07	0.90	0.89(0.04)
LS flex	0.74	0.85	0.06	0.22	0.27(0.06)	0.02	0.87	0.90(0.04)

**Fig. 9:**
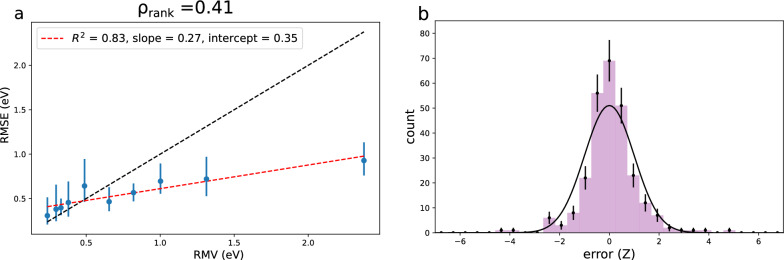
**a** Error-based calibration plot for the epistemic evidential uncertainties one of the vertical IP models. **b** Distribution of errors according to their *Z*-value for the model split with random seed = 42 compared with a Gaussian distribution of width 1. Error-bars are Poisson.We see that the non-zero miscalibration area is mostly caused by an increased number of low errors compared to what is expected from the uncertainty-distribution. This is also clear from the error-based calibration plot, where we observe that it is the errors for the high uncertainties that are significantly lower than what is expected from the corresponding uncertainties, i.e. these uncertainties are overestimated

As has been the case in other studies comparing uncertainty evaluation metrics across different models and data sets, the observed magnitude of the evaluation metrics change from what we observed for the logP dataset (Tables 3 and Additional file 1: S13). While the linear fit for the RMSE/RMV correlation is decent ($R^2=0.83$), the observed slope of 0.27 is much too low and the intercept of 0.35 eV too high. Again we turn to the error-based calibration plots with bootstrapped 95% confidence intervals to get some more insight into the performance of the uncertainty estimates (Fig. 9). The lower uncertainties are quite well-calibrated, but the higher uncertainties are greatly overestimated.

The Spearman’s rank correlation coefficient ($\rho _{rank}=0.41$) is higher than what we observed for any of the uncertainty estimates for the logP data. Meanwhile, $\rho _{rank}^{sim}$ is also much higher ($\rho _{rank}^{sim}=0.56\pm 0.05$) reflecting the uncertainty distribution being much wider in terms of the model RMSE. The fact that the simulated value is higher than the observed one, makes sense based on the error-based calibration plots; the difference in RMSE between high and low uncertainty points is not at all as big as expected from the uncertainty estimates, since the high uncertainty points are severely overestimated. This is an example of an uncertainty estimate with good performance for low uncertainties, while the magnitude of the uncertainties becomes questionable in the >1eV range.

The miscalibration area ($A_{mis}$) is in the higher range of what we have observed so far. The $A_{mis}$ metric is based on the assumption that the distribution of *Z*-errors (that is errors in fractions of the corresponding uncertainty) is Gaussian distributed (width=1). Figure 9b shows the *Z*-distribution for the errors/uncertainties of this model, which clearly does not follow the Gaussian distribution. Note however, that according to the Var$(Z) \overset{?}{=}\ 1$ test, these uncertainties are accepted as being average calibrated ($\langle$ Var$(Z)\rangle = 1.03$, Additional file 1: Table S8)).

Since the optimum values of both NLL and Spearman’s rank correlation coefficient are highly dependent on the nature of the test set and model itself (which defines the test set uncertainty and error distributions), the information that can be gained from these metrics in themselves is limited. This highlights the need for suitable reference values (NLL$^{sim}$ and $\rho _{rank}^{sim}$). However, in this case the observed NLL is within one standard deviation of the simulated value and does imply any problem with the uncertainties.

Again, the by far most informative metrics on the uncertainties were based on the error-based calibration plots.

#### Error-based re-calibration

An option to get better calibrated errors for the evidential epistemic uncertainties is to tune the hyperparameter, $\lambda$. $\lambda$ is a parameter in the evidential loss function determining to what degree it should be prioritized that evidence is lowered for high-error points during training (see Additional file 1: Section S2 or Amini et al. [13] for details on the evidential loss function). As an example, results for $\lambda =0.1$ is presented in section S3, but for a more detailed analysis of the effect of $\lambda$ for evidential models see [28]. Another option is to re-calibrate the uncertainties post training. A popular approach for such re-calibration is a linear re-calibration where the linear parameters are determined by minimizing the NLL on the uncertainties+errors (under assumption of Gaussian errors) of the validation set. This approach was used for the latent space uncertainties and has been applied in several UQ studies [1, 3, 6, 12]. We propose another simple option, that does not assume Gaussian errors, which is to simply use the fitted slope and intercept from a RMSE/RMV plot of the validation set to re-calibrate the uncertainties according to:

8 $$\begin{aligned} \sigma _{cal} = \text {slope}_{val}\cdot \sigma + \text {intercept}_{val} \end{aligned}$$

**Fig. 10:**
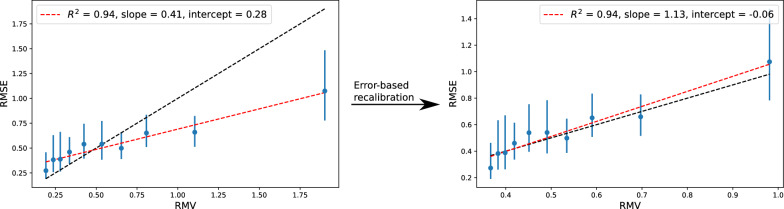
Before and after re-calibration of the model with training data split into training and validation set with random seed = 19 and $\lambda =0.1$

As this is also a linear re-calibration, we will only get good calibrated uncertainties, if there is a strong linear correlation between RMSE and RMV. Figure 10 shows an example of the error-calibration plot before and after re-calibration according to the validation set following Eqn. 8 for one of the models with $\lambda =0.1$ (random seed = 19 for the training data split). After the re-calibration, the RMSE vs RMV plot is in agreement with perfect error-based calibration (all 95% confidence intervals of the RMSE bins overlap the identity line).

For comparison, we also train some simple feed forward NNs with the same training-validation splits as for the evidential models, and analyse the LS uncertainties (details on model architecture in Additional file 1). The RMSE is similar to that of the evidential models (Additional file 1: Table S14). The uncertainty distribution fitted with Eqn. 6 have similar problematic behaviour as we observed for the LS-GCN$_{150\text {k}}$ uncertainties for the logP regression models (e.g. $\rho _{rank} > \rho _{rank}^{sim}$ and slopes generally too high, see Additional file 1: Section S4). Here, we focus on the uncertainties fitted with the more flexible linear relationship (Eqn. 7) with UQ evaluation metrics are summarized in Table 3.

**Fig. 11:**
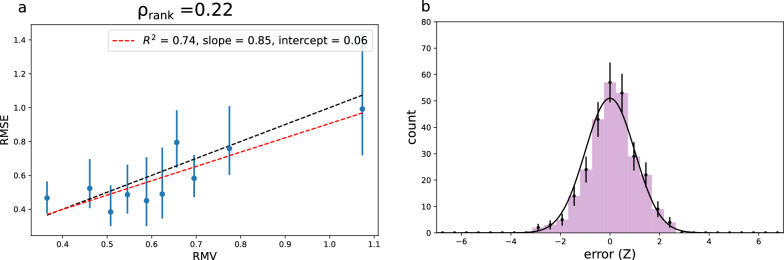
**a** Error-based calibration plot for the LS uncertainties (fitted with Eqn. 7) of one of the five vertical IP models. **b** Distribution of errors according to their *Z*-value for the model split with random seed = 42 compared with a Gaussian distribution of width 1. Error-bars are Poisson

In this case, the linear fit is decent but not great ($R^2$=0.74). Note that some of the models with different train/validation splits show much better linear correlation with $R^2$-values of 0.96 and 0.97 (Additional file 1: Fig. S6). The slope and intercept are both pretty good (close to 1 and 0, respectively and from the error-based calibration plots (Figs. 11a and Additional file 1: Fig. S6) we do generally see good local calibration. In particular, unlike most of the uncertainty estimates analyzed above, we do not see systematic over or underestimated uncertainties. This is also reflected in the Spearman’s rank correlation coefficient ($\rho _{rank}=0.22$) which is in good agreement with the simulated value ($\rho _{rank}^{sim}=0.27\pm 0.06$).

The miscalibration area is significantly lower than for the evidential uncertainties (0.02 vs 0.07). The Var$(Z) \overset{?}{=} 1$ test is also in agreement with average calibration. Comparing the *Z*-distribution from the random seed = 42 models (Figs. 9b and 11b), we see that in this case the LS *Z*-errors are in much better agreement with a Gaussian distribution. This explains the better agreement between Var(*Z*) and $A_{mis}$ since $A_{mis}$ is based on the assumption that errors are Gaussian. Again, the NLL is within one standard deviation of the simulated value, consistent with good overall uncertainties.

One can easily imagine a situation like this, where two models perform similarly based on the test set RMSE, but very differently w.r.t the uncertainty-error distribution. Which model to choose then depends on a consideration of the intended application. If the goal is to get a sample of predictions with as low error as possible, one might choose a model with uncertainties performing similarly to those in Fig. 9a even if it shows bad calibration for higher uncertainties. If one is looking for a more “general purpose” uncertainty estimator one might go with something like those showed in Fig. 11a.

## Design of the test set

**Fig. 12:**
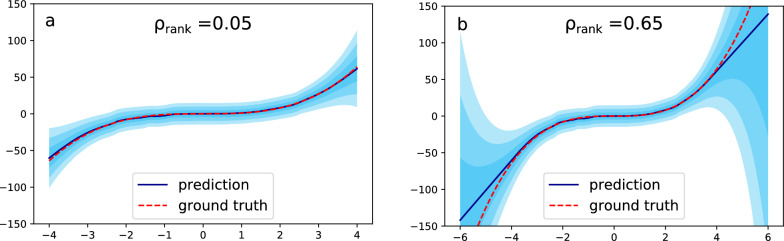
Results for model trained on 50,000 samples ($X_{train} \in [-4,4]$). The shaded area represents the epistemic uncertainty. **a** test set of 1000 samples uniformly sampled from $X_{test} \in [-4;4]$, $\rho _{rank}^{sim}$ = 0.27±0.03, **b** test set of 1000 samples uniformly sampled from $X_{test} \in [-6;6]$ $\rho _{rank}^{sim}$ = 0.65±0.02

From the above examples, it should be apparent that the distribution of test set uncertainties is a key factor determining what we can expect from the UQ validation metrics. Especially ranking-based UQ validation metrics are sensitive to the uncertainty distribution, e.g. for the models presented here we observed $\rho _{rank}^{sim}$ values ranging between 0.04 (practically no correlation expected) and 0.57. A high $\rho _{rank}$ is only possible if the test set uncertainties are well separated (wide distribution). In other words, there should be a big difference in the model performance across different parts of the test set. Thus, if one wants to be able to test the ability of the UQ estimates to distinguish between very high and very low uncertainty predictions, it is important to think about the design on the test set. Many ML models in chemical research are still trained and tested based on a random split of some data set. Assuming a relatively homogeneous data set we should not expect an especially big difference in model performance across the test set. How to design training and test set in order to test e.g. the ability of the model to generalize is subject to increasing attention in the chemical ML literature and we expect similar considerations on test set design for UQ validation to be important going forward.

Here, we illustrate the effect of how the test set is designed on a ranking-based method such as $\rho _{rank}$ through a simple toy example introduced in the original deep evidential regression paper by Amini et al. [13]. Amini et al. tested the performance of their epistemic evidential uncertainties by training a model on $y = x^3 + \epsilon$, with $\epsilon$ drawn from a Gaussian error distribution with standard deviation 3 representing the aleatoric uncertainty. They train the evidential model in the range $X_{train} \in [-4; 4]$ but test in the range $X_{test} \in [-6;6]$ resulting in a very convincing plot (Fig. 3 in [13]) showing how the uncertainty (and error) increases outside the training interval, where the model is forced to extrapolate.

Similarly, we train a model with $X_{train} \in [-4;4]$ but use the same model on two different test sets; one with samples drawn within the training set range [-4;4] and one with samples drawn in the range [-6;6] as in the original study where 1/3 of the test samples are outside of the training set interval. Figure 12 illustrates the different situations and are accompanied by the corresponding Spearman’s rank correlation coefficients (0.05 vs 0.65). Note that while $\rho _{rank}^{sim}=0.65\pm 0.02$ is in good agreement for the test set containing out-of-distribution (OOD) samples, that is not the case for the test set within the training distribution ($\rho _{rank}^{sim}=0.27\pm 0.03$) which indicates that the uncertainties within the training distribution but close to the border are overestimated. This clearly illustrates the effect of how the test set is designed on ranking-based metrics; if we want to test the UQ method’s ability to distinguish between high and low uncertainty prediction we have to design the test set accordingly. Another point should also be noted here; a method for testing the ability of a model to generalize is to compare model error for an in-distribution test set with an OOD test set. Success is in this scenario as little difference in test error/model accuracy as possible. However, for uncertainty predictions we *should* expect a change in the ranking-based metrics as we add OOD data points to the test set.

## Conclusions and outlook

The ability to assign confidence to predictions is becoming an increasingly important aspect of molecular ML. Therefore, the ability to evaluate whether the uncertainty estimates of an uncertainty quantification (UQ) method are reasonable is required. We present a comparison of the error-based calibration method recently proposed by Levi et al. [12] and three popular methods for the evaluation of uncertainty estimates; Spearman’s rank correlation coefficient, the miscalibration area and the negative log likelihood (NLL). We apply the evaluation metrics to uncertainty estimates for two different chemical datasets and three different UQ methods and find the error-based calibration to be the superior choice for UQ evaluation. For the NLL and Spearman’s rank correlation coefficient specifically, we found that the introduction of simulated reference values was necessary for these metrics to bear any meaning.

In agreement with previous studies, we found quite varying performance across validation metric, target property and method [3]. However, while several sets of uncertainty estimates did not perform optimally, all uncertainty estimates studied here possessed valuable information. The important part is then to be aware of the limitations and strengths and for this we found the error-based calibration plots to be an extremely powerful tool for getting detailed information of the uncertainty estimates. In fact most conclusions obtained from the remaining metrics could be drawn directly from analyzing the error-based calibration plot.

Though Gaussian errors are typically assumed (importantly both NLL and miscalibration area relies on this), we found multiple examples of non-Gaussian error distributions, which can lead to different conclusions for average calibration depending on what metric is looked at (e.g. the miscalibration area ($A_{mis}$) or the variance of the *Z*-score, that is error as a fraction of its uncertainty (Var(*Z*))).

In cases of bad calibration (either average or local) one can do a re-calibration based on a validation set. Typically this is done by minimizing the NLL for the validation set. The NLL assumes normally distributed errors, so as an alternative we propose a re-calibration based on the linear error-based calibration fit of the validation set.

While good uncertainty estimates can hold extremely useful information about the degree of trust one should put in a ML prediction, they have yet to become a standard part of chemical ML studies. We believe one of the reasons for that is the lack of consensus on how to best benchmark the uncertainty estimates. One part of reaching that consensus is using an appropriate metric as described above. Another part is figuring out how best to design test sets that actually test the performance of the uncertainties on the intended objective.

One of the important use-cases for uncertainty estimates is in sequential learning applications such as Bayesian optimization and active learning. While several studies on sequential learning applied to chemical datasets have emerged [8, 29], it is often concluded that a greedy search strategy works best i.e. one that ignores the uncertainty estimates. One question to ask is then whether these observations are due to the search strategy itself or due to problems in the calibration of the uncertainty estimates. Consider using the uncertainty estimates presented in Fig. 9a for active learning. The highest uncertainties are greatly overestimated compared to the lower/medium uncertainties. These molecules would be over-sampled to a degree that is unwarranted when considering the actual expected error. The first step towards answering the question above is to have a proper way of evaluating ones uncertainty estimates. We believe the work presented herein will provide a starting point for chemical ML users for incorporating uncertainties in their work in an informed manner.

### Supplementary Information

**Additional file 1**. Additional figures and tables.

## Data Availability

Models and data used in the study can be found here: https://sid.erda.dk/sharelink/dNF1IjDPQB. Code for model training and calculation of evaluation metrics is available here: https://github.com/jensengroup/UQ_validation_methods. For convenience, we have prepared a Colab link where it is possible to upload a.csv file with errors and uncertainties and easily obtain the presented metrics: https://colab.research.google.com/drive/1Jgm9XJvWHQJwWaF_GyDZpEbU_Kk1SSPb
