# Supplementary material for: Uncertain of uncertainties? A comparison of uncertainty quantification metrics for chemical data sets
Source: J Cheminform. 2023 Dec 18;15:121. doi: 10.1186/s13321-023-00790-0 (PMC10729461; doi:10.1186/s13321-023-00790-0)
Supplement: Supplementary file 1 — Additional file 1. Additional figures and tables. [file 13321_2023_790_MOESM1_ESM.pdf]

Supporting Information

S1 Miscalibration area

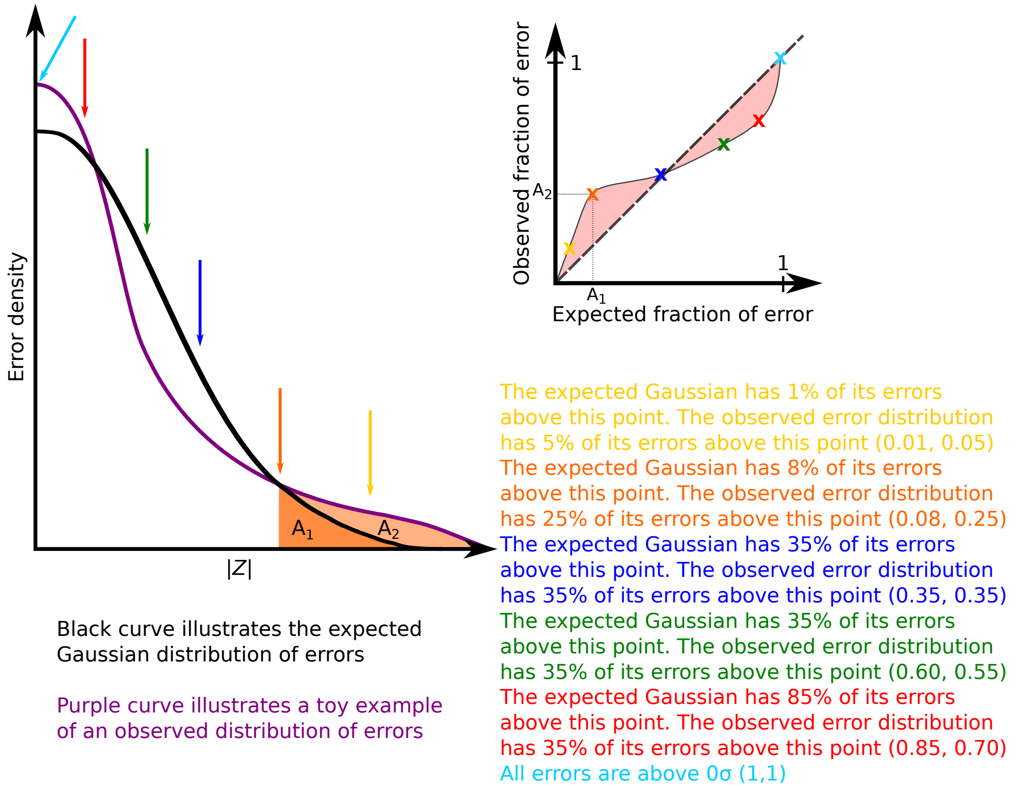

Figure S1: Sketch of how the miscalibration area is obtained

Figure S1 illustrates how the miscalibration area is obtained. The fraction of errors above e.g. 3σ for the observed error distribution (purple) is compared with the fraction of errors expected for a Gaussian distribution of errors (black curve). Comparing these two fraction of errors for different numbers of σ results in a plot of observed vs. expected fraction of errors. If the observed errors fit perfectly with what we expect for Gaussian distributed errors, this plot will be a diagonal. The miscalibration area is then the area between the ideal diagonal and the curve observed.

S2 Model details

RF (scikit-learn: RandomForestRegressor)

- 200 trees
- Minimum number of samples per leaf is three
- Molecular representation; ECFP4 with a binary vector of length 2048

GCNN (PyTorch Geometric)

- Input atom feature vector; 1-hot encoding of atomic number, hybridization, formal charge, chirality, number of hydrogen neighbors and number of heavy atom neighbors. Binary encoding of whether atom

is in ring of not and if it aromatic or not. Scaled numeric values of atomic mass, van der Waals radius and covalent radius. Length of atomic feature vector: 79

- Three GCN layers (GraphConv) each with 300 hidden nodes and with ReLU activation functions.
- Pooling layer combining the atomic feature vectors by calculating the average producing a learned molecular feature vector of size 300.
- A feed-forward NN following the pooling layer with three hidden layers each consisting of 300 nodes in each, with ReLU activation functions and a dropout rate of 0.2.
- Learning rate = 10^{-4}
- Batch size = 128
- Loss = mean squared error (MSE)
- Early stopping with a patience of 30 epochs is used.

Feed-forward NN (PyTorch)

- Input molecular features: revised auto-correlations (RACs[25]) in addition to the oxidation state, spin multiplicity and total charge of the ligands. RACs that are invariant over the dataset are eliminated leaving 154 input features.
- Both input features and output targts are scaled according to the training data using the StandardScaler of scikit-learn.
- Three hidden layers with 300 nodes in each, ReLU activation and dropout rate of 0.2.
- Output layer with a single node and no activation function.
- Batch size = 64
- Loss = MSE
- Learning rate = 10^{-4}
- Early stopping with a patience of 30 epochs is used.

Feed-forward Evidential NN (PyTorch)
The weights, $\mathbf{w}$, of the evidential NN are trained by minimizing the evidential loss function[13]:

$$L_i^{evi}(\mathbf{w}) = L_i^{NLL}(\mathbf{w}) + \lambda \cdot L_i^{R}(\mathbf{w}) \tag{9}$$

$$L_i^{NLL}(\mathbf{w}) = \frac{1}{2}\log\left(\frac{\pi}{\nu}\right) - \alpha \cdot \log(2\beta(1+\nu)) + (\alpha + \frac{1}{2}) \cdot \log((y_i - \gamma)^2\nu + 2\beta(1+\nu)) + \log\left(\frac{\Gamma(\alpha)}{\Gamma(\alpha + \frac{1}{2})}\right) \tag{10}$$

$$L_i^{R}(\mathbf{w}) = |y_i - \gamma| \cdot (2\nu + \alpha) \tag{11}$$

γ is the predicted mean for the property and ν, α and β define predictions for the aleatoric uncertainty ($\frac{\beta}{\alpha-1}$) and epistemic uncertainty ($\frac{\beta}{\nu(\alpha-1)}$). Γ is the gamma function and λ is a hyperparameter controlling how much it should be prioritized that large-error samples have less evidence. The terms of the loss function represents two objectives; L_i^{NLL} optimizes model fitness while L_i^{R} penalizes predictions with high error by lowering evidence. The relative importance of the two terms is controlled by λ. We use $\lambda = 0.2$, which is the default suggested by Soleimany et al. [7]. The epistemic (model) uncertainty is then obtained as

$$\sigma^{epistemic} = \sqrt{\frac{\beta}{\nu \cdot (\alpha - 1)}} \tag{12}$$

- Input molecular features: revised auto-correlations (RACs[25]) in addition to the oxidation state, spin multiplicity and total charge of the ligands. RACs that are invariant over the dataset are eliminated leaving 154 input features.
- Both input features and output targts are scaled according to the training data using the StandardScaler of scikit-learn.
- Three hidden layers with 300 nodes in each, ReLU activation and dropout rate of 0.2.
- Output layer with four nodes; One for the target prediction (γ) with no activation function, two for ν and β with a softplus activation function and one for α with a softplus activation function with 1 added to the output.
- Batch size = 64
- Loss = Evidential loss
- Learning rate = 10^{-4}
- Early stopping with a patience of 30 epochs is used.

S3 Evidential Regression Model with $\lambda = 0.1$

Seed	1	19	27	42	94
RMSE (eV)	0.57	0.57	0.58	0.60	0.67

Table S1: Test set RMSE for the five evidential NN models trained with different random splits of the vertical IP training data and with $\lambda = 0.1$.

seed	NLL	NLL^{sim}	ρ_{rank}	ρ_{rank}^{sim}	A_{mis}	R^2	a	b
1	0.80	0.80(0.04)	0.37	0.51(0.04)	0.06	0.90	0.46	0.24
19	0.85	0.70(0.05)	0.35	0.56(0.04)	0.04	0.94	0.41	0.28
27	0.85	0.76(0.04)	0.39	0.53(0.05)	0.04	0.85	0.46	0.26
42	0.87	0.87(0.04)	0.29	0.51(0.05)	0.06	0.83	0.50	0.22
94	0.95	1.03(0.05)	0.34	0.48(0.05)	0.06	0.85	0.51	0.22

Table S2: UQ evaluation metrics for the evidential uncertainties of the five evidential NN models trained with different random splits of the vertical IP training data and with $\lambda = 0.1$.

seed	Var(Z)	CI(Var(Z))	μ(Z)	CI(μ(Z))
1	1.00	[0.79;1.33]	0.00	[-0.12;0.12]
19	1.29	[1.01;1.78]	-0.03	[-0.16;0.11]
27	1.18	[0.95;1.54]	0.04	[-0.09;0.17]
42	0.98	[0.77;1.31]	0.15	[0.03;0.27]
94	0.82	[0.65;1.11]	0.13	[0.02;23]

Table S3: Z-metrics for the five evidential NN models trained with different random splits of the vertical IP training data and with $\lambda = 0.1$.

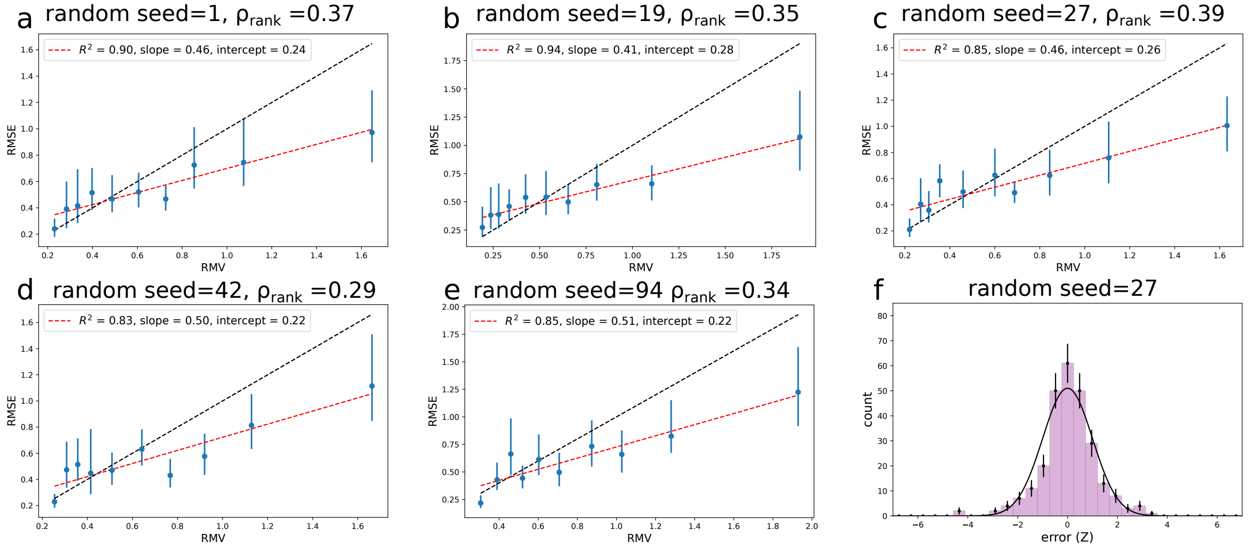

Figure S2: (a-e) Error-based calibration plots for the epistemic evidential uncertainties of the five vertical IP models trained with $\lambda = 0.1$. f) Distribution of errors according to their Z-value for the model split with random seed = 42 compared with a Gaussian distribution of width 1. Error-bars are Poisson.

S4 Latent space uncertainties for vertical IP NN fitted with Eqn. 6

seed	NLL	NLL^{sim}	ρ_{rank}	ρ_{rank}^{sim}	A_{mis}	R^2	a	b
1	0.87	0.99(0.04)	0.21	0.13(0.06)	0.06	0.79	1.15	-0.18
19	0.87	0.91(0.04)	0.28	0.14(0.06)	0.03	0.97	2.21	-0.74
27	0.90	0.96(0.04)	0.32	0.15(0.06)	0.02	0.74	1.47	-0.33
42	0.86	0.89(0.05)	0.22	0.14(0.06)	0.02	0.76	1.67	-0.41
94	0.90	1.01(0.04)	0.25	0.14(0.06)	0.06	0.95	1.80	-0.61

Table S4: UQ evaluation metrics for the LS uncertainties of the five feed forward NN models trained with different random splits of the vertical IP training data.

seed	Var(Z)	CI(Var(Z))	μ(Z)	CI(μ(Z))
1	0.77	[0.65;0.93]	0.03	[-0.08;0.13]
19	0.92	[0.77;1.12]	0.04	[-0.08;0.15]
27	0.86	[0.72;1.03]	0.20	[0.09;0.31]
42	0.93	[0.79;1.13]	0.08	[-0.04;0.20]
94	0.76	[0.63;0.92]	0.17	[0.07;0.28]

Table S5: Z-metrics for the LS uncertainties of the five feed forward NN models trained with different random splits of the vertical IP training data.

S5 Tests for $\mathrm{Var(Z)} \stackrel{?}{=} 1$ and $\mu(\mathrm{Z}) \stackrel{?}{=} 0$

Table S6 shows results for the $\mathrm{Var}(Z) \stackrel{?}{=} 1$ and $\mu(Z) \stackrel{?}{=} 0$ tests. CI(Var(Z)) and CI($\mu(Z)$) are the 95% confidence interval for the variance and average of Z, respectively. The lack of average calibration is clear for the models trained on 100 and 500 data points (Var(Z)>1 meaning underestimated uncertainties) in agreement with our findings based on the error-based calibration plots, miscalibration areas and NLL vs. NLL^{sim}. $\mu(Z)$ is above 0 for the three models trained on the least amount of data indicating biased models, the remaining models are in agreement with unbiased models. The uncertainties from the 5k, 10k, 20k and 50k models all fail the $Var(Z) \stackrel{?}{=} 1$ test for average calibration. This explains the higher-than-expected NLL (by around 5 standard deviations) for the 5k, 10k and 20k models (Table 1). The discrepancy between NLL and NLL^{sim} is less significant for the 50k model and it is also closer to passing the $Var(Z) \stackrel{?}{=} 1$ test. In this case, the fact that there is a problem with average calibration for these four models was not obvious from the miscalibration area, A_{mis}.

Almost all models show a bias, but whether the bias is positive or negative varies between models. For biased errors, the $\mathrm{Var}(Z) \stackrel{?}{=} 1$ changes to the $\langle Z^2 \rangle \stackrel{?}{=} 1$ test for average calibration[5]. The change from Var(Z) to $\langle Z^2 \rangle$ amounts to the addition of $\langle Z \rangle^2$ (zero for unbiased errors). The most biased errors based on Z-scores are those of LS-NN$_{150k}$ for which $\langle Z^2 \rangle = 1.02$ and the 95% confidence interval is [0.96;1.16] consistent with average calibrated errors. Likewise for the remaining models, we see that all models passing the $\mathrm{Var}(Z) \stackrel{?}{=} 1$ test also pass the $\langle Z^2 \rangle \stackrel{?}{=} 1$ test. Though there is some correlation between the uncertainty estimates showing highest miscalibration area ($A_{mis} = 0.07$) and the 4 sets of uncertainty estimates failing the $\langle Z^2 \rangle \stackrel{?}{=} 1$ test (LS-NN$_a$, LS-GCN$_a$, LS-GCN$_b$ and LS-GCN$_d$), there generally is a lack of agreement between the two metrics on what uncertainties have better average calibration. For example, LS-NN$_d$ just passes the $\langle Z^2 \rangle \stackrel{?}{=} 1$ test but still has a miscalibration area of 0.07 which is similar to the RF uncertainties trained on 100 samples

N_{train}	Var(Z)	CI(Var(Z))	μ(Z)	CI(μ(Z))
100	1.47	[1.41;1.54]	0.27	[0.23;0.30]
500	1.24	[1.19;1.29]	0.12	[0.09;0.15]
1,000	1.03	[0.99;1.08]	0.06	[0.04;0.09]
5,000	1.13	[1.08;1.18]	0.00	[-0.03;0.03]
10,000	1.14	[1.09;1.19]	0.00	[-0.03;0.03]
20,000	1.10	[1.06;1.15]	-0.01	[-0.04;0.02]
50,000	1.06	[1.02;1.11]	-0.01	[-0.04;0.01]
100,000	1.05	[1.00;1.10]	-0.02	[-0.05;0.01]
150,000	1.04	[1.00;1.09]	-0.02	[-0.05;0.01]

Table S6: Z-metrics for the nine RF models trained to predict Crippen's logP. The confidence intervals are obtained using the bootsrap method.

LS	Var(Z)	CI(Var(Z))	μ(Z)	CI(μ(Z))
NN_a	0.92	[0.87;0.99]	-0.04	[-0.07;-0.02]
NN_b	0.95	[0.89;1.03]	0.17	[0.14;0.19]
NN_c	1.01	[0.95;1.09]	-0.19	[-0.21;-0.16]
NN_d	0.93	[0.87;1.00]	0.14	[0.12;0.17]
NN_e	1.06	[1.00;1.14]	-0.03	[-0.06;0.00]
NN_{150k}	0.92	[0.86;1.05]	0.32	[0.30;0.35]
GCN_a	0.92	[0.87;0.99]	-0.05	[-0.08;-0.02]
GCN_b	0.89	[0.84;0.97]	0.16	[0.13;0.19]
GCN_c	1.00	[0.95;1.07]	-0.18	[-0.21;-0.15]
GCN_d	0.89	[0.84;0.96]	0.14	[0.11;0.17]
GCN_e	1.06	[0.99;1.13]	-0.03	[-0.06;0.00]
GCN_{150k}	0.90	[0.83;1.01]	0.26	[0.23;0.29]

Table S7: Z-metrics for the five GCNN models trained on different sets of 9k+1k Crippen's logP training data (labelled a-e) where latent space uncertainties are either last layer in the fully connected NN (labelled NN_i) or last layer of the GCN (labelled GCN_i. Metrics for the GCNN model trained on 145k+5k training data is also shown (NN_{150k} and GCN_{150k}). The "a" model corresponds to the N_{train} = 10k model described in the manuscript.

that greatly failed the $\mathrm{Var}(Z) \stackrel{?}{=} 1$ (and $\langle Z^2 \rangle \stackrel{?}{=} 1$) test.

seed	Var(Z)	CI(Var(Z))	$\mu(Z)$	CI($\mu(Z)$)
1	0.96	[0.74;1.28]	0.13	[0.01;0.25]
19	0.79	[0.62;1.052]	0.03	[-0.08;0.13]
27	0.70	[0.57;0.89]	0.10	[0.00;0.20]
42	1.03	[0.79;1.41]	0.04	[-0.08;0.16]
94	0.81	[0.64;1.12]	0.06	[-0.05;0.17]

Table S8: Z-metrics for the five evidential NN models trained with different random splits of the vertical IP training data.

For four out of five models, the 95% confidence interval for the variance of Z is in accordance with the uncertainties being average calibrated (Table S8), but the width of the confidence intervals have increased

significantly for this 265-sample test set. The miscalibration area is calculated based on an assumption of Gaussian distributed errors, while the $\text{Var}(Z) \stackrel{?}{=} 1$ test does not assume that[5]. Thus, the miscalibration area test evaluates the agreement of the Z-distribution being Gaussian with variance = 1, while the $\text{Var}(Z)$ tests if the Z-distribution has variance = 1, but make no assumption on the distribution.

seed	Var(Z)	CI(Var(Z))	μ(Z)	CI(μ(Z))
1	0.79	[0.66;0.96]	0.03	[-0.07;0.14]
19	0.94	[0.80;1.12]	0.06	[-0.06;0.17]
27	1.12	[0.84;2.05]	0.25	[0.13;0.39]
42	0.93	[0.78;1.12]	0.09	[-0.03;0.21]
94	0.82	[0.69;1.01]	0.19	[0.08;0.30]

Table S9: Z-metrics for the LS flex uncertainties of the five feed forward NN models trained with different random splits of the vertical IP training data.

For the LS uncertainty of the NN model, only the model with random seed = 1 failts the $\text{Var}(Z) \stackrel{?}{=} 1$ test for average calibration (Table S9).

S6 Additional tests

S6.1 logP

Model	a	b	c	d	e	150k
RMSE	0.28	0.26	0.31	0.28	0.29	0.16

Table S10: Test set RMSE for the five GCNN models ($a-e$) trained on different sets of 9k+1k Crippen's logP training data as well as the 145k+5k model.

LS	R^2	a	b	ρ_{rank}	ρ_{rank}^{sim}	A_{mis}	NLL	NLL^{sim}
NN$_a$	0.31	1.12	-0.05	-0.04	0.07(0.01)	0.07	0.14	0.17(0.01)
NN$_b$	0.31	0.73	0.06	-0.03	0.13(0.01)	0.06	0.08	0.09(0.01)
NN$_c$	0.49	0.88	0.04	0.01	0.09(0.01)	0.03	0.24	0.21(0.01)
NN$_d$	0.47	1.18	-0.06	-0.02	0.09(0.01)	0.07	0.12	0.15(0.01)
NN$_e$	0.24	1.33	-0.09	-0.04	0.04(0.01)	0.05	0.18	0.15(0.01)
NN$_{150k}$	0.91	1.20	-0.03	0.17	0.24(0.01)	0.03	-0.50	-0.51(0.01)
GCN$_a$	0.24	0.65	0.09	-0.02	0.11(0.01)	0.07	0.14	0.18(0.01)
GCN$_b$	0.47	0.99	-0.01	0.01	0.10(0.01)	0.07	0.08	0.12(0.01)
GCN$_c$	0.44	1.28	-0.08	-0.01	0.06(0.01)	0.04	0.24	0.22(0.01)
GCN$_d$	0.51	1.54	-0.17	0.01	0.07(0.01)	0.07	0.13	0.17(0.01)
GCN$_e$	0.39	1.65	-0.18	-0.03	0.05(0.01)	0.05	0.18	0.15(0.01)
GCN$_{150k}$	0.85	1.85	-0.13	0.23	0.13(0.01)	0.05	-0.46	-0.45(0.01)

Table S11: UQ evaluation metrics for the latent space uncertainties of a GCNN model trained on 9k+1k data points of Crippen's logP. For NN uncertainties, the latent space used is the very last layer of the NN. For GCN uncertainties, the latent space is the vector right after the pooling layer (LS-GCN).

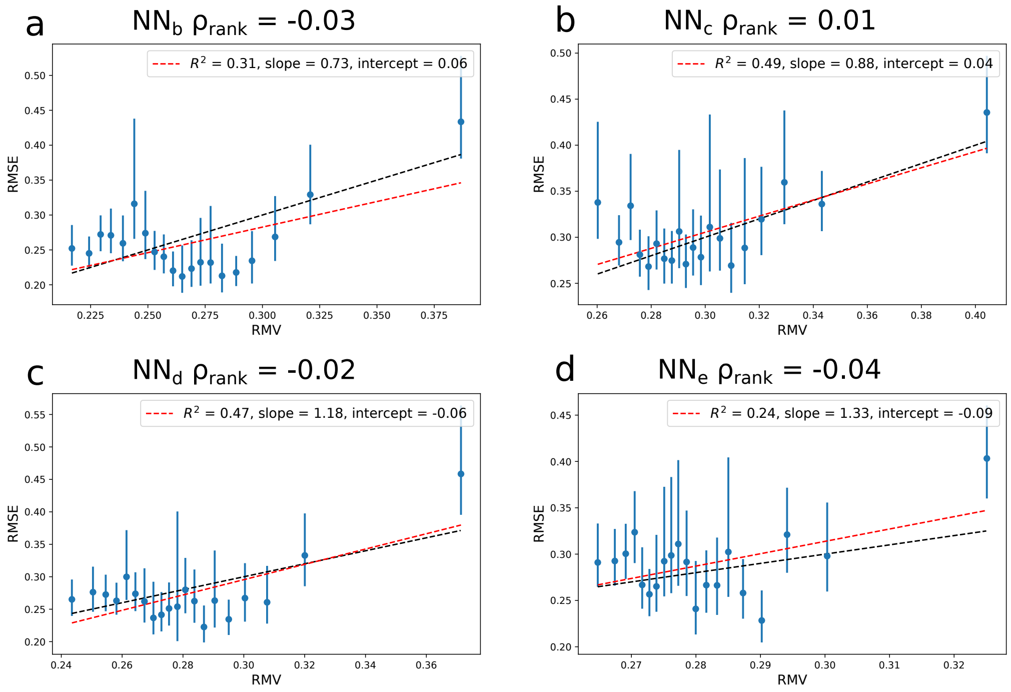

Figure S3: Error-based calibration plots for the LS-NN uncertainties based four GCNN models trained on different sets of 9k+1k Crippen's logP training data (labelled a-e) as well as the GCNN model trained on 145k+5k samples. Each bin contains 250 of the test samples. Error bars on the RMSE bins represent 95% confidence intervals and are found by bootstrapping.

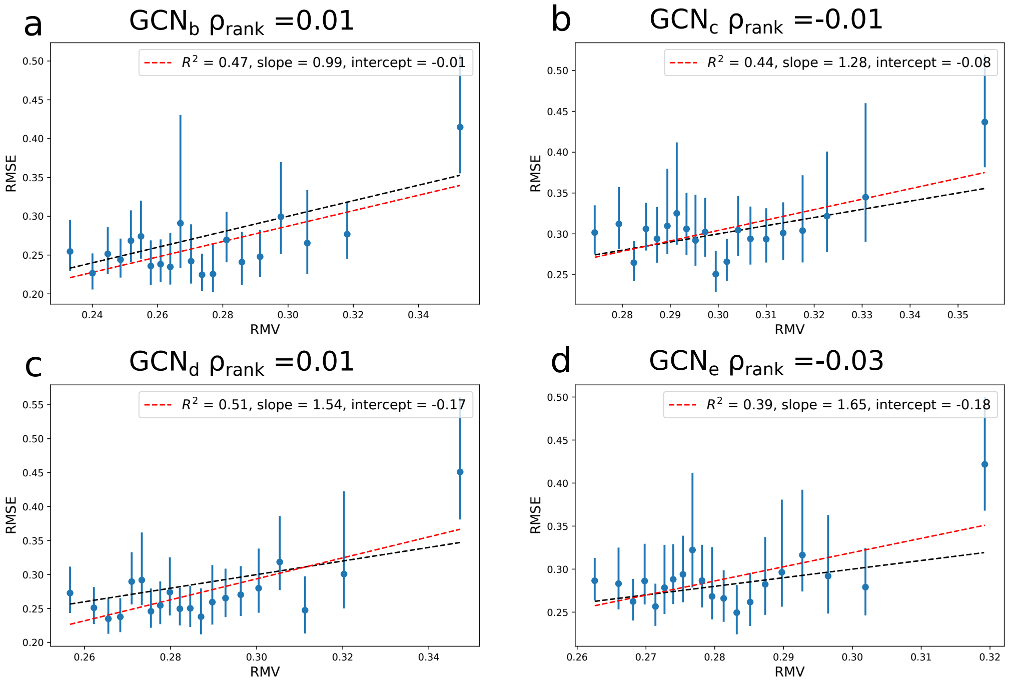

Figure S4: Error-based calibration plots for the LS-GCN uncertainties based four GCNN models trained on different sets of 9k+1k Crippen's logP training data (labelled a-e) as well as the GCNN model trained on 145k+5k samples. Each bin contains 250 of the test samples. Error bars on the RMSE bins represent 95% confidence intervals and are found by bootstrapping.

S6.2 Vertical IP

Seed	1	19	27	42	94
RMSE (eV)	0.55	0.59	0.65	0.58	0.59

Table S12: Test set RMSE for the five evidential NN models trained with different random splits of the vertical IP training data.

seed	R^2	a	b	ρ_{rank}	ρ_{rank}^{sim}	A_{mis}	NLL	NLL^{sim}
1	0.58	0.21	0.37	0.41	0.57(0.04)	0.07	0.85	0.87(0.04)
19	0.89	0.30	0.31	0.37	0.55(0.05)	0.10	0.85	0.96(0.04)
27	0.81	0.44	0.23	0.37	0.51(0.05)	0.09	0.92	1.06(0.04)
42	0.83	0.27	0.35	0.41	0.56(0.05)	0.07	0.90	0.89(0.04)
94	0.55	0.22	0.37	0.39	0.56(0.04)	0.09	0.94	1.03(0.04)

Table S13: UQ evaluation metrics for the evidential uncertainties of the five evidential NN models trained with different random splits of the vertical IP training data.

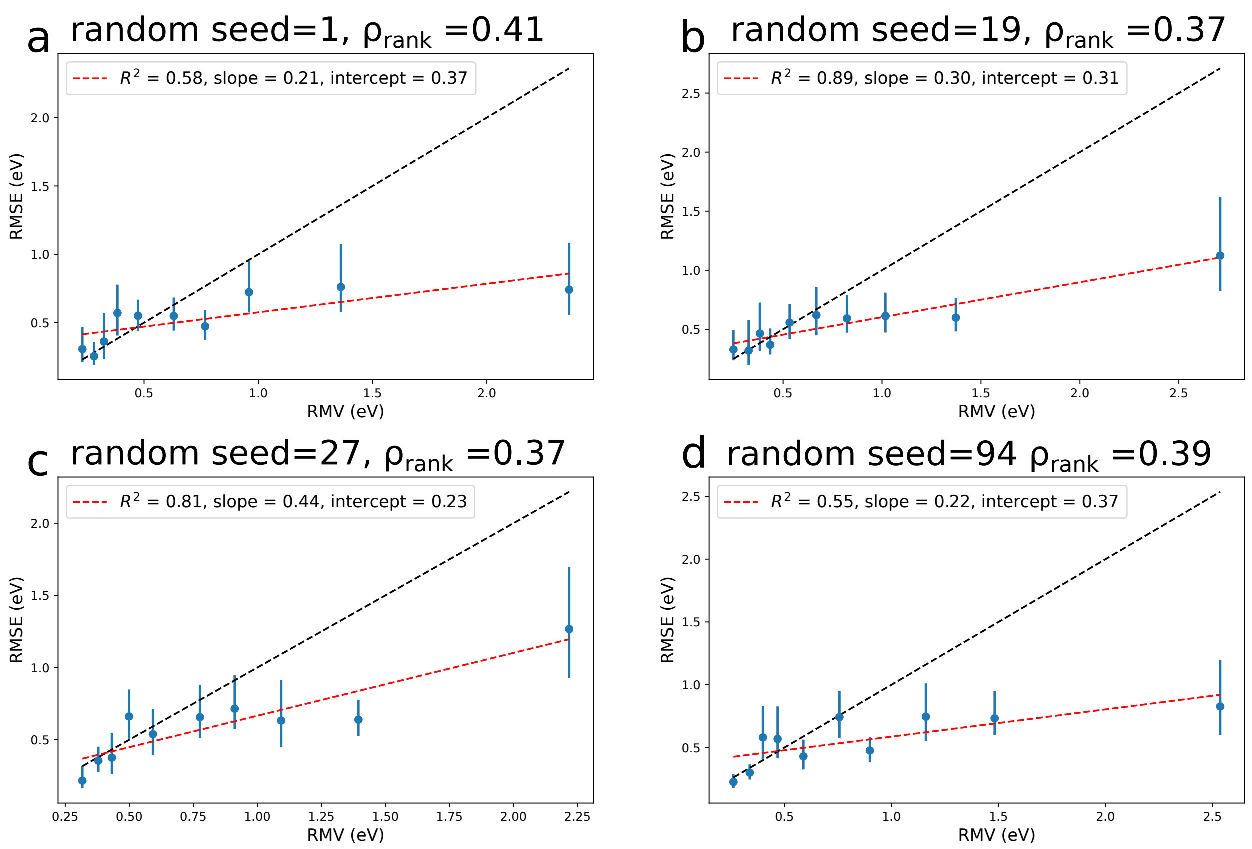

Figure S5: Error-based calibration plots for the epistemic evidential uncertainties of the four additional vertical IP models.

Seed	1	19	27	42	94
RMSE (eV)	0.59	0.64	0.63	0.61	0.64

Table S14: Mean test set RMSE for the five feed foward NN models trained with different random splits of the vertical IP training data.

seed	R^2	a	b	ρ_{rank}	ρ_{rank}^{sim}	A_{mis}	NLL	NLL^{sim}
1	0.81	0.66	0.13	0.21	0.24(0.06)	0.06	0.87	0.98(0.04)
19	0.97	1.26	-0.16	0.28	0.26(0.06)	0.02	0.85	0.88(0.04)
27	0.76	0.79	0.10	0.32	0.29(0.05)	0.02	1.01	0.92(0.04)
42	0.74	0.85	0.06	0.22	0.27(0.06)	0.02	0.87	0.90(0.04)
94	0.96	0.98	-0.05	0.25	0.27(0.06)	0.05	0.89	0.97(0.04)

Table S15: UQ evaluation metrics for the LS flex uncertainties of the five feed forward NN models trained with different random splits of the vertical IP training data.

The problems with the average calibration for the model with random seed 1 can also be inferred by observing the error-based calibration plot; all but one of the binned RMSE values are below that predicted based on the RMV (black dashed line) pointing to a tendency of overestimated uncertainties leading to a both lower than expected NLL and uncertainties that are not average calibrated.

The NLL values are similar to those for the evidential model as expected for models with similar accuracy. The NLL is generally in good agreement with the simulated one (the biggest discrepancy is for the model split with random seed 1, which is also the model with highest miscalibration area).

The miscalibration areas are generally lower than those from the evidential model and the agreement between A_{mis} and $\mathrm{Var}(Z)$ is better.

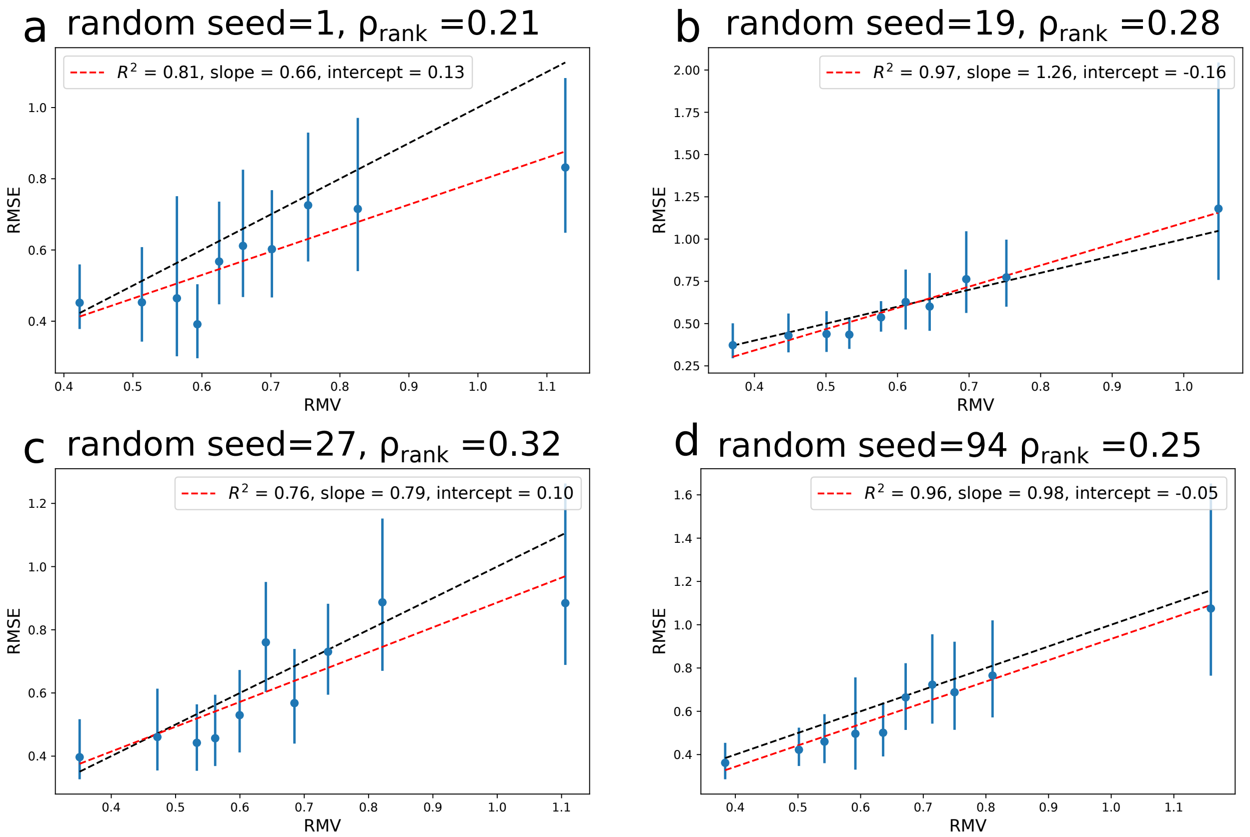

Figure S6: Error-based calibration plots for the LS uncertainties (fitted with Eqn. 7) of the four vertical IP models.
